# How to Form Behavioral Intentions in the Field of Drone Food Delivery Services: The Moderating Role of the COVID-19 Outbreak

**DOI:** 10.3390/ijerph17239117

**Published:** 2020-12-06

**Authors:** Jinsoo Hwang, Dohyung Kim, Jinkyung Jenny Kim

**Affiliations:** 1 The College of Hospitality and Tourism Management, Sejong University, Seoul 143-747, Korea; jhwang@sejong.ac.kr; 2 Seoulland F&B, 181, Gwangmyeong-ro, Gwacheon-si, Gyeonggi-do 13829, Korea; dhkim@seoulland.co.kr; 3 School of Hotel and Tourism Management, Youngsan University, 142 Bansong Beltway, Haeundae-gu, Busan 48015, Korea

**Keywords:** drone food delivery services, COVID-19, overall image, desire, behavioral intentions

## Abstract

This study was designed to identify the significance of drone food delivery services using the moderating role of the outbreak of COVID-19. More specifically, this study proposed that there is a positive relationship between the overall image and the desire. Additionally, it was hypothesized that the desire helps to enhance two types of behavioral intentions, which included word-of-mouth intentions and the willingness to pay more. Lastly, the moderating role of the outbreak of COVID-19 was proposed during this process. Six hypotheses were tested that used 335 samples before the outbreak of COVID-19, and 343 samples were used after the outbreak of COVID-19 in South Korea. The data analysis results indicated that the overall image has a positive influence on the desire, which in turn positively affects the word-of-mouth intentions and the willingness to pay more. Furthermore, this study identified the important moderating role of the outbreak of COVID-19 in the relationship between the desire and the word-of-mouth intentions.

## 1. Introduction

The coronavirus disease, which will hereafter be referred to as COVID-19, is a viral respiratory disease that first broke out in Wuhan, China in December 2019 [1]. As of September 2020, there were more than 33,492,000 confirmed cases and more than 1,005,000 COVID-19 deaths globally [2]. Around the world, this tragic pandemic has had a significant effect on the restaurant industry. For instance, according to the National Restaurant Association [3], about 8 million people, which is about two-thirds of the people working in the foodservice industry, have lost their jobs since the COVID-19 outbreak. Also, a sales loss of about US$ 240 billion is expected by the end of 2020.

One of the reasons these negative statistical results are predicted is that people do not want to eat out, because they want to avoid coming into contact with the COVID-19 infection. This means that after the COVID-19 outbreak, the consumers have tended to prefer food delivery services at home rather than eating out [4]. In this situation, drone food delivery services, which are a contactless service, are attracting attention in the foodservice industry, because the services provide food without face-to-face encounters [5].

Drones are one of the fast-growing sectors recently, and their applications are widely increasing in diverse industries due to the significant utilization values in many aspects. For example, drones are used for agricultural imaging, emergency healthcare, product distribution, traffic monitoring, and transportation [6]. Among these various usages, using drones for deliveries is worth attempting from a pro-environment standpoint. Drone home deliveries are being researched by a number of companies, such as Amazon, UPS, and Walmart as an alternative to delivering products using trucks [7]. More specifically, drones consume less fuel and have a smaller impact on the environment, which therefore makes drone delivery one of the most environment-friendly transportation options [8]. Yoo et al. [9] asserted that drone delivery is faster, less expensive, and more eco-friendly than traditional delivery facilities, such as trucks and motorbikes.

The foodservice industry is not spared from the drones’ invasion. Uber has been approved by the U.S. federal government for food delivery tests using drones, which began in San Diego [10]. Uber could begin delivering food using drones in 2021 [11]. The company pointed out that the customers could receive food at their doorsteps in five to thirty minutes. Uber is not the first company to attempt food delivery using drones. FoodPanda offers a delivery service using drones in 20 min in Hong Kong [10], and Dominos is the first big food chain to test food delivery using drones. Various benefits of the drone food delivery services were identified through a series of experiments, and the environmental role of drone-based food delivery has particularly been highlighted in the existing literature [5]. This literature illustrates that many companies are capitalizing on the commercial value of drone food delivery services, which is based on its superior role regarding environmental protection.

Despite the importance of drone food delivery services after the coronavirus pandemic, the research on it is insufficient. Thus, this study tried to examine how to form behavioral intentions in the context of drone food delivery services. More specifically, the objectives of this study are to examine (1) the effect of the overall image of drone food delivery services on the desire to use the services, (2) the influence of the desire to use drone food delivery services on the behavioral intentions, which includes the word-of-mouth intentions and the willingness to pay more, and (3) the moderating role of the COVID-19 outbreak during this process.

## 2. Literature Review

### 2.1. Overall Image

The concept of the overall image has been consistently studied in diverse fields, which include airlines, hotels, restaurants, and tourism [12,13,14,15]. The overall image can be defined as one of the customers’ characteristics regarding a product/service [16]. Similarly, Assael [17] also suggested that the overall image refers to the overall recognition of a certain product/service formed based on information obtained from various sources of the product/service. In other words, the overall image of a product/service can be created based on its detailed attributes [18]. The overall image of a product/service is considered a critical factor that affects the customers’ decision-making processes [12,19]. In addition, the overall image plays an important role to improve corporate profits, so many companies are making efforts to create a positive overall image [20,21].

Many previous studies have explored the antecedents and consequences of the overall image in diverse fields. For instance, Han, Kiatkawsin, and Kim [22] tried to examine how to form the overall image and its effect on outcome variables using 325 samples in the hotel industry. They showed that emotional experiences play an important role in the formation of the overall image. In addition, they also suggested that the overall image has a positive influence on satisfaction. Sharma and Nayak [23] developed a research model to find the antecedents and consequences of the overall image based on the 398 samples in the tourism industry. Their data analysis results indicated that the overall image is formed by cognitive and affective images. Furthermore, they revealed that the overall image helps to enhance behavioral intentions.

### 2.2. Effect of Overall Image on Desire

Next, this paper proposed the effect of the overall image on the desire. In the hospitality and tourism industry, the concept of desire, which was derived from the model of the goal-directed behavior (MGB), has been studied to identify consumer behavior, which can be defined as “a state of mind whereby an agent has a personal motivation to perform an action or to achieve a goal” [24] (p. 71). The desire is a state that arises through an internal stimulus that triggers an action for a certain purpose, which includes achievements, curiosity, and shortages [24,25].

More importantly, one of the most significant factors with forming desire is the overall image of a product/service [26], which means that when consumers have a favorable image of a certain product/service, they would have a high level of desire. The empirical research also has supported the relationship between the overall image and the desire. For instance, Hwang and Lyu [25] developed a research model to find the relationship between the image and the desire using 320 samples in the airline industry. Their data analysis results indicated that the image is a significant factor in forming the desire. In addition, Han et al. [27] examined the relationship between the image and the desire using 258 customers in the field of screen golf. They found the effect of the image on the desire and suggested that when customers have a positive image of screen golf, they are more likely to use screen golf. In addition, Han and Hyun [28] investigated how the overall image affects the desire based on 286 passengers in the cruise industry. They showed that the overall image is an important factor that affects desire. Integrating the theoretical and empirical backgrounds, the following hypothesis was developed.

**Hypothesis** **1** **(H1).** *The overall image has a positive influence on the desire.*

### 2.3. The Effect of the Desire on the Behavioral Intentions

The behavioral intentions refer to the likelihood that an individual will engage in a particular behavior [29], and they consist of the following two concepts [30,31,32] that include (1) the word-of-mouth intentions and (2) the willingness to pay more. First, the concept of word-of-mouth intentions can be defined as “informal, person to person communication between a perceived non-commercial communicator and a receiver regarding a brand, a product, an organization or a service” [33] (p. 63). Consumers are more likely to rely on information from other people, which includes family members and friends, more than commercial advertisements [34,35]. Second, the willingness to pay more is defined as the maximum amount the consumers intend to pay for their preferred brand of products compared to the other brands [36], so it is important to find out how to enhance the willingness to pay more from the corporate point of view, which leads to an enhancement of the corporate profits [37,38].

According to the MGB, when consumers try to engage in a certain behavior, they tend to show high levels of behavioral intentions [39], which indicates that there is a positive relationship between the desire and the behavioral intentions. The prior studies have also suggested the effect of the desire on behavioral intentions. For example, Han et al. [40] examined the role of desire in the formation of behavioral intentions in the field of medical hotels, and they suggested that desire positively affects behavioral intentions. In addition, Hwang and Choe [41] also investigated the relationship between desire and behavioral intentions. They found that desire is a key factor that affects behavioral intentions in the field of drone food delivery services. More recently, Lee et al. [42] tried to investigate the relationship between desire and behavioral intentions using 320 samples in the tourism industry. They showed that desire aids to increase behavioral intentions. Based on the theoretical and empirical backgrounds, the following hypotheses can be proposed.

**Hypothesis** **2** **(H2).** *The desire has a positive influence on word-of-mouth intentions.*

**Hypothesis** **3** **(H3).** *Desire has a positive influence on the willingness to pay more.*

### 2.4. The Moderating Effect Before and After the Outbreak of COVID-19

After the outbreak of COVID-19, the consumers have been reluctant to have interactions with others because of the infectious nature of COVID-19 [43], so they prefer contactless services [44]. This phenomenon also has had a great influence on the consumers’ perceptions of drone food delivery services, because the services are provided without face-to-face contact with the consumers [5]. In addition, according to Bauer [45], the potential risk has a great impact on consumer behavior. In other words, the consumers’ perceptions of drone food delivery services can be different according to the perceived risks such as the outbreak of COVID-19.

Many prior studies have shown the significance of perceived risk in consumer behavior. For example, Wells et al. [46] found that perceived risks negatively affect the relationship between perceived novelty and attitude in the technology industry. In addition, Ahmed et al. [47] showed the moderating role of perceived risk in the relationship between attitude and behavioral intentions in the field of online shopping. Kim, Jang, and Kim [48] indicated that food technology neophobia plays an important moderating role in the relationship between attitude and behavioral intentions.

In terms of before and after the outbreak of COVID-19, the empirical studies also examined the changes in consumer behaviors in the foodservice industry. For instance, Long and Khoi [49] found changes in the consumption patterns, which includes the hoarding of groceries by the consumers, due to the risk of COVID-19, which is based on the theory of planned behavior (TPB). In addition, Manivannan et al. [50] explained the importance and the growth of the online food delivery industry as a result of the changes in the consumers’ buying behaviors after the outbreak of COVID-19. Jain [51] also argued that an important change in consumer patterns in the restaurant industry was predicted after the outbreak of COVID-19.

Drone food delivery services are considered as a representative case of contactless services [52,53]. In addition, Southey [4] suggested that the COVID-19 outbreak significantly affects consumer behavior related to contactless food delivery services. McFarland [54] also argued that drones are an original and efficient solution because the drones guarantee “zero human-contact” when delivering. As the importance of contactless services increases, the consumers’ perceptions of drone food delivery services can change before and after the outbreak of COVID-19, which means the moderating effect before and after the outbreak of COVID-19.

**Hypothesis** **4** **(H4a).** *The relationship between the overall image and the desire is significantly moderated by the outbreak of COVID-19.*

**Hypothesis** **4** **(H4b).** *The relationship between the desire and the word-of-mouth intentions is significantly moderated by the outbreak of COVID-19.*

**Hypothesis** **4** **(H4c).** *The relationship between the desire and the willingness to pay more is significantly moderated by the outbreak of COVID-19.*

**Figure 1:**
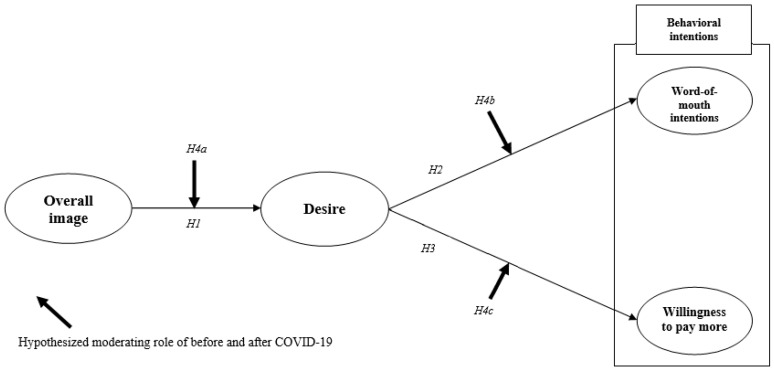
The proposed conceptual model.

Based on the theoretical relationships between the constructs, the following research model is proposed (Figure 1).

## 3. Methodology

### 3.1. Measures

To measure the five constructs, which include the overall image, the desire, the word-of-mouth intentions, and the willingness to pay more, this study cited multi items that were proved to be reliable and valid in the previous studies (see Appendix A). First, the overall image was measured using three items that were adapted from Han et al. [12] and Jani and Han [55]. Second, the desire was measured using three items that were used by Han and Yoon [56] and Perugini and Bagozzi [39]. Third, the measures for the word-of-mouth intentions were borrowed from Hennig-Thurau, Gwinner, and Gremler [57]. Fourth, the willingness to pay more was measured using three items that were cited from Han et al. [12]. A seven-point Likert scale that ranged from strongly disagree (1) to strongly agree (7) was used to measure all the items in this study.

### 3.2. Data Collection

In this study, two surveys were conducted before and after the COVID-19 outbreak to identify the moderating role of the outbreak of COVID-19. To collect data, this study employed the convenience sampling technique using an online survey. First, the data collection before the COVID-19 outbreak was collected for research other than the purpose of this study. In South Korea, drone food delivery services have not been commercialized yet, so respondents were given about 2 min and 30 s of the video to enhance their understanding of the services before the survey (see Appendix B). The video explained the operation system of drone food delivery services well. In addition, the system was set so that respondents can join the survey after watching the video. The first survey was based on the survey system of EMBRAIN, which is one of the biggest market research companies in South Korea. The company sent an e-mail invitation to 2794 perspective participants for three days in February 2018, and 346 of them joined the survey. In addition, it is widely known that outliers can lead to wrong statistical results [58,59], so 11 outliers were deleted after checking visual inspections and multivariate outliers. Consequently, 335 samples were used for further statistical analysis.

Second, another data collection was conducted, which used the same data collection method as the first data collection, and was performed after the outbreak of COVID-19 in South Korea. As with the first data collection, respondents were shown the same video to enhance their understanding of drone food delivery services before the survey. The online survey company sent 1479 emails over three days in May 2020, and 343 of them completed the survey. As a result, 343 samples were employed for further statistical analysis.

### 3.3. Profile of Survey Respondents

Table 1 shows the profile of the survey respondents. First, in regards to before the outbreak of COVID-19, there were 194 males (57.9%) and 141 females (42.1%). In addition, 37.6% of the respondents (*n* = 126) were in their 20 s, which was followed by 31.0% in their 30 s (*n* = 104). More than half of the respondents were college graduates (*n* = 197 and 58.8%), and 56.7% of respondents were single (*n* = 190). In regards to the income level, the highest percentage of the respondents earned between US $2001 ~ US $3000 (*n* = 76, 22.7%).

Second, in regards to after the outbreak of COVID-19, there were 177 males (51.6%) and 166 females (48.4%) that participated. Additionally, the largest age group included participants in their 30 s (*n* = 107 and 31.2%), and more than half of the respondents (*n* = 226 and 65.9%) hold a bachelor’s degree. In regards to the marital status, 198 (57.7%) of the participants were single. Lastly, the highest percentage of the respondents earned between US $2001 ~ US $3000 (*n* = 97 and 28.3%).

### 3.4. Confirmatory Factor Analysis (CFA)

The CFA results of for the three models, which included before the outbreak of COVID-19, after the outbreak of COVID-19, and merging the two, showed that the overall fit of the measurement model was statistically adequate (Before the outbreak of COVID-19: χ^2^ = 131.590, df = 48, χ^2^/df = 2.741, *p* < 0.001, NFI = 0.979, CFI = 0.986, TLI = 0.981, and RMSEA = 0.072; After the outbreak of COVID-19: χ^2^ = 14.589, df = 48, χ^2^/df = 2.950, *p* < 0.001, NFI = 0.978, CFI = 0.985, TLI = 0.980, and RMSEA = 0.076; and Merging before and after the outbreak of COVID-19: χ^2^ = 183.973, df = 48, χ^2^/df = 3.833, *p* < 0.001, NFI = 0.985, CFI = 0.989, TLI = 0.985, and RMSEA = 0.065) [60]. In addition, all of the factor loadings were equal to or higher than 0.904 for before the outbreak of COVID-19, 0.936 for after the outbreak of COVID-19, and 0.930 for the merged version. Table 2 illustrates the specific variables employed in this study along with their standardized factor loadings.

As shown in Table 3, the values of all the average variance extracted (AVE) for the three models exceeded 0.50, which supports that all the constructs used in this study had an acceptable convergent validity [61]. In addition, the results revealed that the values of the composite reliabilities of all the constructs for the three models are greater than 0.70, which means that all the constructs used in this study had a satisfactory level of internal consistency [62]. Lastly, the results show that the values of all the AVE for the three models exceeded all of the squared correlations (R2) between any pair of constructs, which suggests that all the constructs for the three models had a high level of discriminant validity [63].

### 3.5. Structural Equation Modeling (SEM)

**Figure 2:**
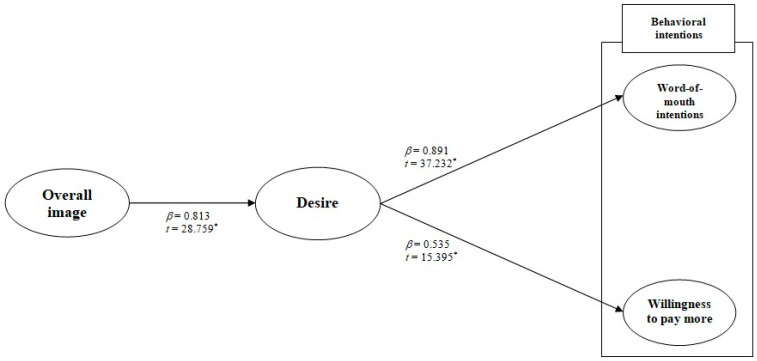
The standardized theoretical path coefficients. Note: * *p* < 0.05.

The proposed hypotheses were checked using an SEM analysis. The overall evaluation of the model fit showed an acceptable fit of the model to the data (χ^2^ = 240.461, df = 51, χ^2^/df = 4.715, *p* < 0.001, NFI = 0.981, CFI = 0.985, TLI = 0.980, and RMSEA = 0.074). Figure 2 presents the results with the standardized coefficients and their t-values. All three proposed hypotheses were statistically supported at *p* < 0.05. More specifically, the overall image has a positive influence on the desire (*β* = 0.813 and *t* = 28.759 *), so Hypothesis 1 was supported. In addition, the desire positively affects both the word-of-mouth (*β* = 0.891 and *t* = 37.232 *) and the willingness to pay more (*β* = 0.535 and *t* = 15.395 *), which supported Hypotheses 2 and 3.

### 3.6. Measurement-Invariance Assessment

According to Steenkamp and Baumgartner [64], this study performed a measurement invariance assessment before testing the moderating role of the outbreak of COVID-19. The samples were divided into two groups, which included before the outbreak of COVID-19 (*n* = 335) and after the outbreak of COVID-19 (*n* = 343). As presented in Table 4, the non-restricted model (χ^2^ = 273.179, df = 96, χ^2^/df = 2.845, *p* < 0.001, NFI = 0.978, CFI = 0.986, TLI = 0.980, and RMSEA = 0.052) and the full-metric invariance model (χ^2^ = 279.758, df = 104, χ^2^/df = 2.689, *p* < 0.001, NFI = 0.978, CFI = 0.986, TLI = 0.982, and RMSEA = 0.050) had adequate fit statistics. In addition, the difference between the two models was not significant (Δχ^2^ = 6.579 < χ^2^ = 0.01(df =15) = 20.090), which means that the full metric invariance was statistically supported.

### 3.7. Moderating Role of the Outbreak of COVID-19

Table 5 presents the results of the moderating role of the outbreak of COVID-19. To test the moderating role of the outbreak of COVID-19, multiple-group analyses were employed by comparing the chi-square difference between the unconstrained models and the constrained models, which was based on the difference in the degrees of freedom [60]. In multiple-group analyses, if the value of the chi-square difference between the unconstrained models and the constrained models is higher than 3.84 (df = 1), it indicates that there is a moderating effect [60].

The data analysis results showed that the outbreak of COVID-19 moderated the relationship between the desire and the word-of-mouth (Δχ^2^ = 5.749 > χ^2^ = 0.5(1) = 3.84, and df = 1), which supported Hypothesis 4b because the value of chi-square difference between the unconstrained models and the constrained models is greater than 3.84 (df = 1). More specifically, the path coefficient for the group after the outbreak of COVID-19 (*β* = 0.917 and *t* = 26.237 *) was higher than the path coefficient for the group before the outbreak of COVID-19 (*β* = 0.820 and *t* = 19.526 *). That is, after the outbreak of COVID-19, consumers are more likely to say positive things about drone food delivery services to others when they have high levels of desire than before the outbreak of COVID-19.

However, Hypotheses 4a (Δχ^2^ = 0.070 < χ^2^ = 0.5(1) = 3.84, and df = 1) and 4c (Δχ^2^ = 0.101 < χ^2^ = 0.5(1) = 3.84, and df = 1) were not statistically supported because the values of chi-square difference between the unconstrained models and the constrained models are less than 3.84 (df = 1). In other words, there is no moderating effect.

## 4. Discussion and Implications

First, the data analysis results showed that the overall image has a positive influence on the desire (*β* = 0.813 and *p* < 0.05). It can be interpreted that when consumers perceived that the overall image of using drone food delivery services is good, their desire to use the services is strong. The results of this study are consistent with the prior studies [26,27], which suggested that the overall image positively affects the desire. This study further extended the important role of the overall image with the formation of the desire in the field of drone food delivery services. The results also have the following managerial implications. It is widely known that the overall image of a certain product is formed by its detailed attributes [18]. In this respect, it is recommended to emphasize the innovative roles and the eco-friendly roles of drone food delivery services. For example, drone food delivery services have the advantage of being able to quickly deliver food that is ordered by the customers regardless of the time and the location, because drones move in the air [5]. In addition, unlike motorcycles and cars, drone food delivery services are operated by batteries, which help to protect the environment [19]. Therefore, if restaurant companies emphasize the impressive features of drone food delivery services, the consumers would have a good image of the services.

Second, the results of the data analysis indicated that the desire positively affects the two dimensions of behavioral intentions, which include the word-of-mouth intentions (*β* = 0.891 and *p* < 0.05) and the willingness to pay more (*β* = 0.535 and *p* < 0.05). This means that when the consumers’ desire to use drone food delivery services when ordering food is strong, they are more likely to recommend the services to others or pay more for the services. Many prior studies have consistently shown the importance of desire [26,27,28]. In this regard, this confirmed the significant role of the desire in the field of drone food delivery services, which is considered a significant theoretical implication of this study.

Lastly, this study found the moderating role of the outbreak of COVID-19 in the relationship between the desire and the word-of-mouth intentions, so hypothesis 4c was supported. More specifically, the path coefficient between the desire and the word-of-mouth intentions for the group after the outbreak of COVID-19 (*β* = 0.917) was greater than the path coefficient between the desire and the word-of-mouth intentions for the group before the outbreak of COVID-19 (*β* = 0.820). The data analysis result can be interpreted that after the outbreak of COVID-19, people are more likely to recommend drone food delivery services to others when they have high levels of desire to use the services than before the outbreak of COVID-19. The results of this study are due to the following reasons. After the outbreak of COVID-19, people tend to prefer contactless services to reduce the risk of infection [52]. As previously explained, drone food delivery services are considered representative contactless services, so people are more inclined to mention the advantages of the services to others after the outbreak of COVID-19. Therefore, if restaurant companies emphasize the role of drone food delivery service as contactless services after the outbreak of COVID-19 through advertisements, people would have high word-of-mouth intentions for the services.

## 5. Limitations and Future Research

The current study has significant theoretical and practical implications, which were mentioned above. However, this study also has the following limitations. First, since the data for this study was collected in South Korea, it is difficult to apply the findings of this study to other regions. Second, even though the delivery services that use drones are used not only in the foodservice industry but also in other industries, the results of this study focused only on the foodservice industry. Therefore, it is necessary to increase the external validity by applying the research model proposed in this study to other industries in future studies. Third, drone food delivery services are not presently activated in South Korea. Therefore, it is recommended to research the customers who have used drone food delivery services in future research. Forth, the current study attempted to find the moderating role of the COVID-19 outbreak. For this reason, the samples are collected at different time periods, which can lead to a bias in the results. Thus, future research needs to collect data at the same time after COVID-19 ends. Lastly, nowadays, drone food delivery services are in the text phase, but the services will be activated worldwide in due course. For this reason, governments around the world are working on regulations for utilizing drones in our lives [65,66]. That is, although there are currently no legal requirements for droned food delivery services, there will be formal legal processes, such as operation and licensing in the future.

## 6. Conclusions

The objective of the current study was to identify how to form behavioral intentions in the field of drone food delivery services. More specifically, this study proposed the effects of the overall image on the desire. In addition, it was hypothesized that the desire helps to enhance the two dimensions of the behavioral intentions, which include word-of-mouth intentions and the willingness to pay more. Lastly, the moderating role of the COVID-19 outbreak was proposed in this process. To evaluate the six hypotheses, this study collected data from 335 samples before the outbreak of COVID-19 and 343 samples after the outbreak of COVID-19 in South Korea. The results of the data analysis have the important theoretical and managerial implications the development of drone food delivery services after the COVID-19 outbreak.

## Figures and Tables

**Table 1:** Profile of the survey respondents.

Variable	Before the Outbreak of COVID-19 (*n* = 335)	After the Outbreak of COVID-19 (*n* = 343)	The Results of the Chi-Square Test	Merging the Data (*n* = 678)
**Gender**				
Male	194 (57.9%)	177 (51.6%)	*p* = 0.058	371 (54.7%)
Female	141 (42.1%)	166 (48.4%)		307 (45.3%)
**Age**				
20 s	126 (37.6%)	103 (30%)	*p* = 0.037 *	229 (33.8%)
30 s	104 (31%)	107 (31.2%)		211 (31.1%)
40 s	70 (20.9%)	102 (29.2%)		172 (25.4%)
50 s	35 (10.4%)	31 (9%)		66 (9.7%)
**Education level**				
Less than High school diploma	35 (10.4%)	30 (8.7%)	*p* = 0.295	65 (9.6%)
Associate’s degree	53 (15.8%)	43 (12.5%)		96 (14.2%)
Bachelor’s degree	197 (58.8%)	226 (65.9%)		423 (62.4%)
Graduate degree	50 (14.9%)	44 (12.8%)		94 (13.9%)
**Marital status**				
Single	190 (56.7%)	198 (57.7%)	*p* = 0.897	388 (57.2%)
Married	142 (42.4%)	141 (41.1%)		283 (41.7%)
Others	3 (0.9%)	4 (1.2%)		7 (1%)
**Income level**				
6001$ US and over	60 (17.9%)	21 (6.1%)	*p* = 0.001 *	81 (11.9%)
5001$ US–6000$ US	37 (11%)	10 (2.9%)		47 (6.9%)
4001$ US–5000$ US	51 (15.2%)	30 (8.7%)		81 (11.9%)
3001$ US–4000$ US	53 (15.8%)	49 (14.3%)		102 (15%)
2001$ US–3000$ US	76 (22.7%)	97 (28.3%)		173 (25.5%)
1001$ US–2000$ US	46 (13.7%)	67 (19.5%)		113 (16.7%)
Under 1000$ US	12 (3.6%)	69 (20.1%)		81 (11.9%)

Note: * *p* < 0.05.

**Table 2:** The confirmatory factor analysis: Items and loadings.

Construct and Scale Item	Standardized Loading ^a^		
	Before the Outbreak of COVID-19	After the Outbreak of COVID-19	Merging Before and After the Outbreak of COVID-19
**Overall image**			
The overall image of using drone food delivery services is good.	0.949	0.936	0.943
The overall image I have about drone food delivery services is great.	0.963	0.954	0.959
Overall, I have a good image about drone food delivery services.	0.919	0.945	0.931
**Desire**			
I desire to use drone food delivery services when ordering food.	0.957	0.947	0.952
My desire to use drone food delivery services when ordering food is strong.	0.963	0.961	0.962
I want to use drone food delivery services when ordering food.	0.962	0.956	0.960
**Word-of-mouth intentions**			
I am likely to say positive things about drone food delivery services to others.	0.958	0.956	0.958
I am likely to recommend drone food delivery services to others.	0.904	0.959	0.930
I am likely to encourage others to use drone food delivery services.	0.962	0.964	0.961
**Willingness to pay more**			
I am likely to pay more to use drone food delivery services.	0.953	0.946	0.950
It is acceptable to pay more to use drone food delivery services.	0.966	0.972	0.969
I am likely to spend extra to use drone food delivery services.	0.973	0.968	0.971
Goodness-of-fit statistics Before the outbreak of COVID-19: χ^2^ = 131.590, df = 48, χ^2^/df = 2.741, *p* < 0.001, NFI = 0.979, CFI = 0.986, TLI = 0.981, and RMSEA = 0.072 After the outbreak of COVID-19: χ^2^ = 14.589, df = 48, χ^2^/df = 2.950, *p* < 0.001, NFI = 0.978, CFI = 0.985, TLI = 0.980, and RMSEA = 0.076 Merging before and after the outbreak of COVID-19: χ^2^ = 183.973, df = 48, χ^2^/df = 3.833, *p* < 0.001, NFI = 0.985, CFI = 0.989, TLI = 0.985, and RMSEA = 0.065			

Notes 1: ^a^ All factors loadings are significant at *p* < 0.001, Notes 2: NFI = normed fit index, IFI = incremental fit index, CFI = comparative fit index, TLI = Tucker–Lewis index, and RMSEA = root mean square error of approximation.

**Table 3:** The descriptive statistics and the associated measures.

	Mean (Std dev.)	AVE	(1)	(2)	(3)	(4)
(1) Overall image	4.48 (1.30)	0.891	0.961	0.761 ^a^	0.71	0.459
	4.45 (1.35)	0.893	0.962	0.762	0.768	0.408
	**4.46 (1.32)**	**0.892**	**0.961**	**0.808**	**0.763**	**0.43**
(2) Desire	4.30 (1.48)	0.924	0.579 ^b^	0.973	0.807	0.509
	4.15 (1.38)	0.911	0.581	0.969	0.784	0.524
	**4.23 (1.43)**	**0.918**	**0.653**	**0.971**	**0.786**	**0.524**
(3) Word-of-mouth	4.54 (1.43)	0.887	0.504	0.651	0.959	0.543
	4.18 (1.32)	0.921	0.59	0.615	0.972	0.618
	**4.36 (1.39)**	**0.905**	**0.582**	**0.618**	**0.966**	**0.576**
(4) Willingness to pay more	3.22 (1.63)	0.929	0.211	0.259	0.295	0.975
	3.07 (1.50)	0.926	0.166	0.274	0.382	0.974
	**3.15 (1.56)**	**0.928**	**0.185**	**0.275**	**0.332**	**0.975**

Notes 1: The unmarked values are for before the outbreak of COVID-19; The underlined values are for after the outbreak of COVID-19, and Values in boldface type are for merging before and after the outbreak of COVID-19. Notes 2: AVE = Average Variance Extracted, Notes 3: Shades. composite reliabilities are along the diagonal, Notes 4. ^a^. correlations are above the diagonal and ^b^. squared correlations are below the diagonal.

**Table 4:** The measurement-invariance models.

	Models	χ^2^	*df*	NFI	CFI	TLI	RMSEA	Δχ^2^	Full-Metric Invariance
Before and after the outbreak of COVID-19	Non-restricted model	273.179	96	0.978	0.986	0.980	0.052	*Δ*χ^2^ (8) = 6.579 *p* > 0.01 (insignificant)	Supported
	Full-metric invariance	279.758	104	0.978	0.986	0.982	0.050		

Notes 1: NFI = Normed Fit Index, CFI = Comparative Fit Index, TLI = Tucker-Lewis Index, and RMSEA = Root Mean Square Error of Approximation. Notes 2: Δχ^2^ (8) = 20.09 and *p* > 0.01.

**Table 5:** The moderating role of the outbreak of COVID-19.

Path	Unconstrained Model				Constrained Model	Tests of Moderator	
	Before the Outbreak of COVID-19		After the Outbreak of COVID-19				
	*β*	*t*-Value	*β*	*t*-Value	*Δ*χ^2^ (102) = 320.396	χ^2^ difference	Hypotheses
H4a OI → D	0.770	18.123 *	0.864	23.663 *	*Δ*χ^2^ (103) = 320.466	*Δ*χ^2^ (103) = 0.07	Not supported
H4b D → WOM	0.820	19.526 *	0.917	26.237 *	*Δ*χ^2^ (103) = 326.145	*Δ*χ^2^ (103) = 5.749	Supported
H4c D → WPM	0.520	10.480 *	0.545	11.157 *	*Δ*χ^2^ (103) = 320.497	*Δ*χ^2^ (103) = 0.101	Not supported

Notes 1: OI = Overall Image, D = Desire, WOM = Word-of-mouth, and WPM = Willingness to pay more. Notes 2: * *p* < 0.05 Notes 3: *_Δ_*χ^2^(1) = 3.84 and *p* < 0.05.

**Figure A1:**
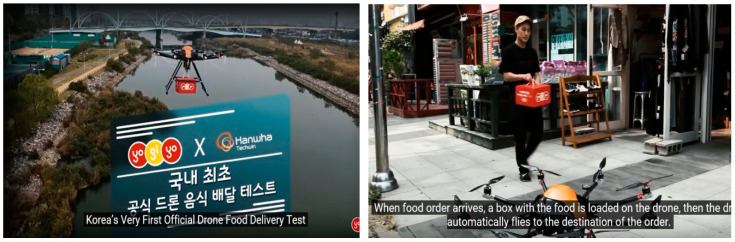
Screenshot from Videos (Source: Yogiyo [67]).

## Appendix A. The Questionnaire

1. Please read each item carefully and circle the appropriate number which best reflects your true opinions or feelings.

Overall Image	Strongly Disagree		→	Neutral	→	Strongly Agree	
The overall image of using drone food delivery services is good.	1	2	3	4	5	6	7
The overall image I have about drone food delivery services is great.	1	2	3	4	5	6	7
Overall, I have a good image about drone food delivery services.	1	2	3	4	5	6	7

2. Please read each item carefully and circle the appropriate number which best reflects your true opinions or feelings.

Desire	Strongly Disagree		→	Neutral	→	Strongly Agree	
I desire to use drone food delivery services when ordering food.	1	2	3	4	5	6	7
My desire to use drone food delivery services when ordering food is strong.	1	2	3	4	5	6	7
I want to use drone food delivery services when ordering food.	1	2	3	4	5	6	7

3. Please read each item carefully and circle the appropriate number which best reflects your true opinions or feelings.

Word-of-Mouth Intentions	Strongly Disagree		→	Neutral	→	Strongly Agree	
I am likely to say positive things about drone food delivery services to others.	1	2	3	4	5	6	7
I am likely to recommend drone food delivery services to others.	1	2	3	4	5	6	7
I am likely to encourage others to use drone food delivery services.	1	2	3	4	5	6	7

4. Please read each item carefully and circle the appropriate number which best reflects your true opinions or feelings.

Willingness to Pay More	Strongly Disagree		→	Neutral	→		Strongly Agree	
I am likely to pay more to use drone food delivery services.	1	2	3	4	5	6		7
It is acceptable to pay more to use drone food delivery services.	1	2	3	4	5	6		7
I am likely to spend extra in order to use drone food delivery services.	1	2	3	4	5	6		7
								
